# Complete Dental Implant Restoration in an Individual With Systemic Sclerosis and Microstomia: A Case Report

**DOI:** 10.1155/2024/9928608

**Published:** 2024-10-23

**Authors:** Alireza Hashemi Ashtiani, Mehrnaz Moradinejad, Vahid Rakhshan

**Affiliations:** ^1^Department of Prosthodontics, School of Dentistry, Ahvaz Jundishapur University of Medical Sciences, Ahvaz, Iran; ^2^Department of Orthodontics, School of Dentistry, Ahvaz Jundishapur University of Medical Sciences, Ahvaz, Iran; ^3^Department of Anatomy, Azad University of Medical Sciences, Tehran, Iran

**Keywords:** dental care for people with special needs, dental implants, microstomia, prosthodontics, systemic scleredema (systemic sclerosis)

## Abstract

**Background:** Systemic sclerosis (SS) is a debilitating disease that affects oral and any other tissues including skin, bone, blood vessels, and the connective tissues by excessive collagen accumulation. It is a difficult case for oral rehabilitation, let alone dental implantation. In this regard, only few studies have been conducted. This article reports a case of full-mouth implant–supported prostheses in a SS patient.

**Case:** After diagnosing most remaining teeth as hopeless through clinical and radiographic examinations, implant-based fixed prosthesis for both jaws was planned, considering the progressive microstomia. Hopeless teeth were extracted. In the maxilla, the areas of central incisors, canines, first premolars, and first molars were implanted. In the mandible, the areas of the lateral incisors and the right second premolar were implanted. Also, according to the surgeon's opinion, the anterior mandible needed bone grafting. After 3 months, the prosthetic treatment was started. Because in scleroderma, the limitation of mouth opening is progressive (and also in order to allow the restoration of the prosthesis in the future), screw-retained abutments were used for the posterior segment. Nevertheless, the anterior abutments were cement-retained.

**Result:** The patient was followed up until the present time (for 3 years). In these follow-ups, no bone resorption was observed, and the treatment was deemed successful.

**Conclusion:** This report suggests that dental implants might be successful and safe for at least some cases of systemic scleredema.

## 1. Introduction

Systemic sclerosis (SS) is referred to a rare chronic disease of connective tissue characterized by fibrosed skin and possibly grave visceral and vascular lesions [1, 2]. The etiology is possibly autoimmune, and the disease onset usually happens between the ages of 45 and 65 or in the third to sixth decades of life [2, 3].

Scleroderma is consisted of localized scleroderma and SS. The former is characterized by skin thickening, stiffening, and vasculopathy and is a result of too much production of collagen and severe skin fibrosis, which occasionally also affects the underlying tissues to the bone [4]. The latter typically starts as Raynaud's phenomenon with fingers growing pale and becoming cyanotic as a result of cold or emotional stress because of vasospasm of unwell blood vessels [5]. SS is rare and has a prevalence of about 1 in every 4000 Americans, much greater than in other countries, with 19 new cases per million detected each year [6] and possibly more common in African-Americans [7]. Females tend to be affected much more than men, with female to male ratios ranging from 3:1 to 14:1 [8]. In Iran, the prevalence of SS is not documented. However, there are reports of characteristics of patients with this disease. For example, according to a study, 68% and 32% of their 200 systematic sclerosis cases were diffuse and limited, respectively; all patients showed the Raynaud's phenomenon; and 91.5% of them were women [9].

SS has numerous dental and orofacial signs as well [2, 3]. These comprise fibrosis of oral mucosal and gingival tissues, xerostomia, dental caries, mask-like look, thin lips, rigidity of tongue and lips, color change of lips and tongue, trismus, muscular atrophy, periodontal problems such as stripping of attached gingiva, gingival recession, periodontal attachment loss, lower gingival bleeding index, PDL widening, a shallower pouch depth, and more large-scale problems such as trigeminal neuralgia, resorption of the angle of the mandible, and resorption of condyle and the coronoid process sometimes even leading to fractures as well as temporomandibular joint disorders [1, 3, 10].

The abovementioned tendency of SS patients to greater and more frequent periodontal damages including attachment loss usually results in early tooth loss. Thus, these patients usually need prosthodontic care. Treatment of such patients has its difficulties such as narrowing of the mouth opening and tongue rigidity which are the most common issues [11]. Prosthodontic care is quite difficult for microstomia patients without surgical assistance, particularly when the mouth perimeter is below 160 mm [11].

The question remaining to be answered completely is whether such patients can be treated properly using dental implants [2, 3, 12]. This article reports implant-supported rehabilitation of a scleroderma patient with microstomia, without any additional surgical treatments.

## 2. Case Presentation

### 2.1. Demographics

The patient was a 39-year-old woman who was referred for restoration of her edentulous areas.

### 2.2. Chief Complaint

Her chief complaint was the mobility of the remaining teeth, esthetic problems, and lack of mastication ability.

### 2.3. Medical History and Systemic Presentation

**Figure 1:**
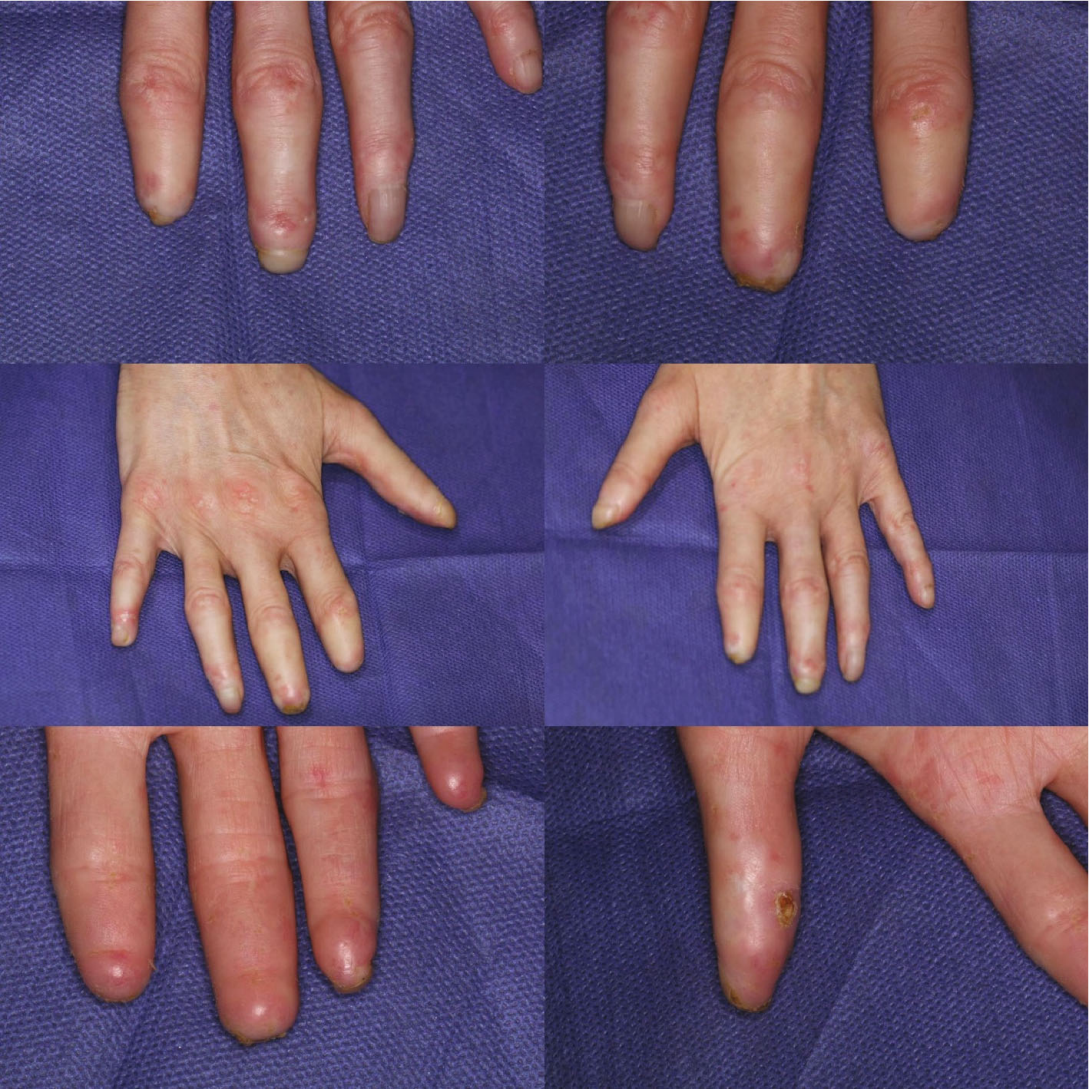
Images of the patient's fingers.

The patient's medical history indicated systemic scleroderma; the condition had caused various complications such as sensitivity to water and cold temperatures, bruising of the legs and hands following even mild degrees of cold temperatures, severe coughing several times a day, difficulty in walking, stiffness of the soles of the feet, and wounds on the fingers (Figure 1) and especially the feet. The disease onset was at the age of 6 years. The patient did not express any family history.

### 2.4. Medications Consumed

The consumed medications included prednisolone, omeprazole, diltiazem, calcium, vitamins D and E, bosentan, and mycophenolic acid.

### 2.5. Current Oral Presentation

**Figure 2:**
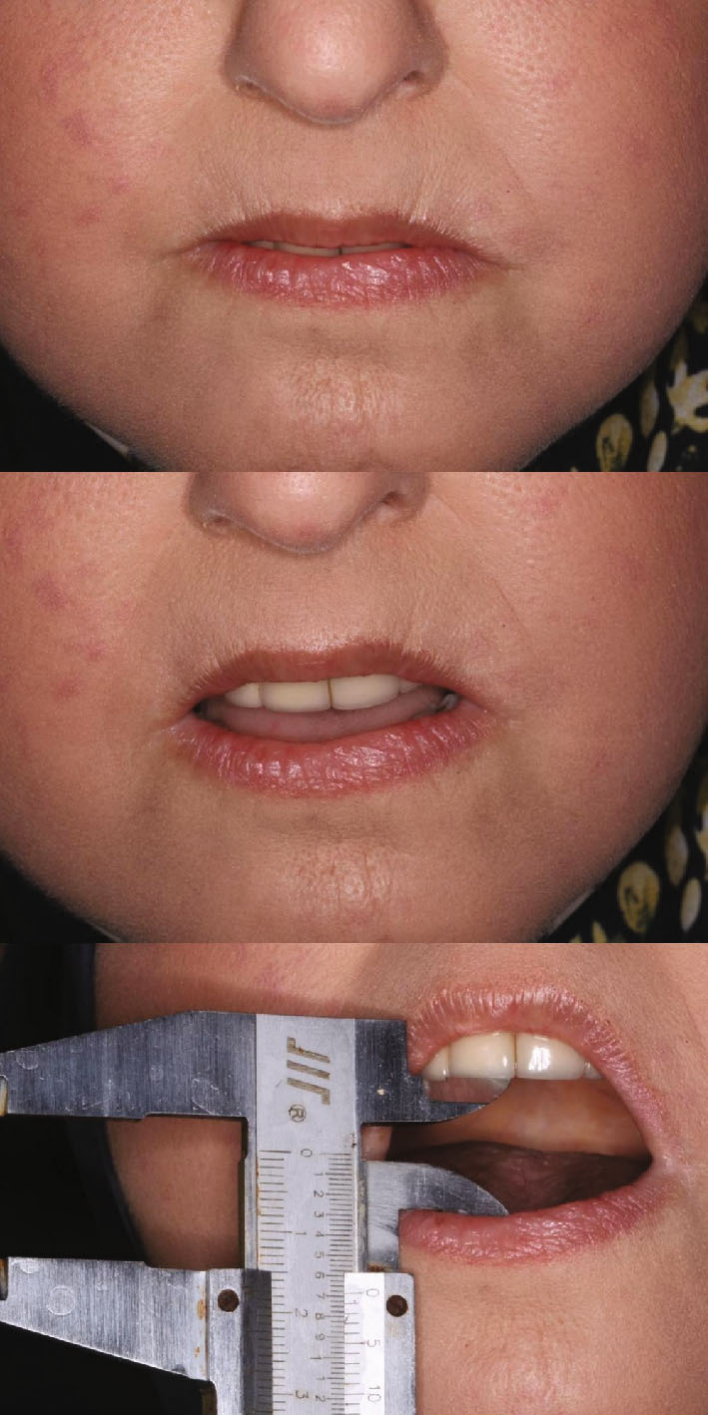
Images of the mouth in closed to maximum opening states.

Upon clinical assessment, microstomia was noted, characterized by a reduced oral aperture and taut oral musculature, with accompanying lip rigidity. The maximum opening of the mouth was 22 mm (Figure 2). The gingivae were thin. The upper and lower anterior teeth had third degree mobility. The prognoses of the bilateral maxillary canines, premolars, and molars were hopeless due to severe caries. The patient profile was mild convex.

### 2.6. Diagnosis and Treatment Planning

First, the patient was referred to a periodontist for further evaluation. The periodontist diagnosed all maxillary teeth, lower laterals, and mandibular right second premolar as hopeless. This was done through examining the amount of bone loss with probing depth and periapical radiographs and examining tooth mobility.

It was possible to provide two treatment plans for this patient: (A) upper and lower removable prosthesis and (B) implant-based fixed prosthesis for both jaws. Considering the progressive limitation of mouth opening and the patient's preference, it was decided to treat the patient with an implant-based fixed prosthesis. CBCT was prepared for this purpose. In the maxilla, the areas of central incisors, canines, first premolars, and first molars were considered for implant placement. In the mandible, the areas of the lateral incisors and the right second premolar were considered for placing the implant. Also, according to the surgeon's opinion, the anterior mandible needed bone grafting.

### 2.7. Treatment

All implants were from ITI (Straumann, Basel, Switzerland). First, the hopeless teeth were extracted because of severe mobility and attachment loss. After initial healing, a temporary full removable denture for the maxilla and a temporary partial removable denture for the mandible were made. After 6 months, the implants (Bone level, ITI, Straumann, Basel, Switzerland) were placed.

All surgeries were free hand and two-staged. No guided surgery planning was done. The patient's transitional removable prosthesis was duplicated and used as a surgical guide to mark the location of the implants. Due to the small size of her mouth, guided surgery was difficult and impractical. In the maxilla, two wide-neck standard implants with a diameter of 4.8 mm and a length of 10 mm were inserted in the first molar region bilaterally. In other areas, regular-neck standard implants with a diameter of 4.1 mm and a length of 12 mm were inserted. In the mandible, in the lateral areas, two 3.3-mm narrow-neck CrossFit implants with a length of 12 mm were inserted, and in the mandibular second premolar area, a standard regular-neck 4.1-mm implant with a length of 10 mm was inserted. In the anterior mandible region, due to insufficient buccal bone thickness and thread exposure, guided bone regeneration (GBR) was performed. For this purpose, three layers of bone material were used: autogenous, allograft, and, at the end, xenograft. A 0.2- to 0.4-mm thick and 15 × 20 mm^2^ pericardial membrane was fixed with two 3-mm pins.

During the healing period after implant placement, the provisional denture was relined every 3 weeks with temporary soft material (TDV Soft Provision, Santa Catarina, Brazil) and used as a temporary denture. A mandibular partial denture was not used because of GBR.

After 3 months, a second surgery was performed and gingival formers were installed. After 2 weeks, the prosthetic treatment was started: Despite the limited opening of the mouth, usual impression taking was possible. Hence, impressions were taken from both jaws using a custom tray and with the open tray method. After preparing the casts, the base and wax rim were prepared, and the centric relation and vertical dimension were recorded. The try-in session was done using the wax rim on which the teeth were mounted. There was no need to increase labial support. As for the patient's smile line level, since the patient was completely edentulous at the beginning of the treatment, the upper smile arch was adjusted with a wax rim.

Artificial acrylic teeth (Yamahachi Dental, Gamagori, Japan) were set according to the wax rims. After verification of the trial dentures in the mouth, an index was prepared with additional silicon (Panasil, Kettenbach, Eschenburg, Germany), which was used for abutment selection.

The anterior teeth were cement-retained due to the direction of the implants, but the posterior teeth were screw-retained due to easier retrievability because of low accessibility of the posterior area. Because in scleroderma, the limitation of mouth opening is progressive, screw-type prostheses were used for the posterior segments in order to allow the retrievability of the prosthesis in the future. The anterior segments were cement-retained due to the position or angulation of implants. For the two posterior maxillary segments, synOcta 1.5 abutments and synOcta plastic copings for bridges were used. In other regions, straight and 15° angled synOcta abutments were used.

The metal framework was made based on the index. It was tested for fitness and passivity in the mouth; it was also checked through radiography. Passivity was confirmed by one-screw test and periapical graph. For the screw-retained segments, one screw test was used. After porcelain application, the prostheses were checked and adjusted in terms of occlusion and esthetics. The patient seemed to be Class III from the beginning because the remaining posterior teeth and canine teeth were in a Class III relationship at the beginning. As the patient was skeletal Class III, crossbite occlusion was established. Uniform occlusal contacts were created and distributed on all the teeth. In the excursive movements, working contacts (group function) made disocclusion on the nonworking side. Finally, the prostheses were polished and glazed.

In the case of cement-retained prostheses, the abutments were tightened to 35 Ncm using a screwdriver and a torque wrench. The screw holes were sealed using Teflon (Dena, Isfahan, Iran), and the prostheses were cemented with zinc oxide eugenol temporary cement (Temp-Bond, Kerr, Italy). The posterior prostheses were also placed and tightened to 15 Ncm. In the posterior segment, the screw holes were sealed using Teflon (Dena, Isfahan, Iran) and composite resin (*Σ* Quick OA2, Tokuyama Dental Corporation, Tokyo, Japan). No multiunits were placed. As much as possible, we tried to maintain posterior disocclusion in lateral movements, and in maximum intercuspation, all teeth had uniform contact.

### 2.8. Outcome

**Figure 3:**
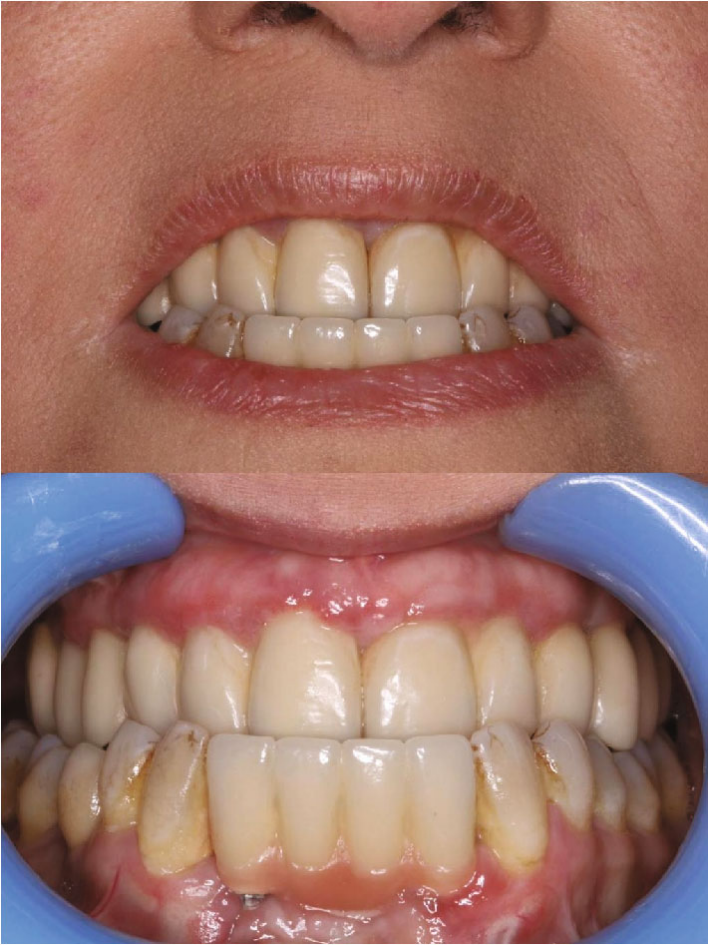
The treatment outcome in a recent follow-up.

The follow-ups were performed in accordance with an implant dentistry textbook [13]. The patient was followed up according to a specific schedule: 3 months posttreatment, 6 months posttreatment, 12 months posttreatment, and then once a year (up to 3 years later [the present time]). In these follow-ups, according to serial parallel periapical radiographies, no bone resorption was observed, and the treatment was deemed successful (Figure 3). Due to finger deformities, plaque control was impaired to some degree and bleeding on probing was observed. Hence, an electrical tooth brush and a waterjet was prescribed. Annual follow-ups will be held for this patient.

## 3. Discussion

The prognosis of dental implants in SS patients may not be ideal. An increased risk of oral mucosa and periodontal diseases, caries formation, and bone quality changes (due to medications) may be seen as medical comorbidities of autoimmune diseases such as scleroderma [14]. Such diseases can also affect the prognosis of dental implantation, due to the vital role of the immune system in at least three areas: (A) the osseointegration process by regulating the host's inflammatory response to dental implants, (B) balancing the marginal bone loss, and (C) the seal of soft tissue around the implant [14]. Nevertheless, clinical evidence suggests otherwise. Despite the lack of any valid guidelines for dental implantation for SS patients [15] and despite the fact that maintaining oral hygiene might be more difficult in SS patients, routine oral rehabilitations using dental implantation have been shown successful over 10 years and more [15, 16]. Such routine implants may be successful and safe for oral rehabilitation of edentulous patients suffering from some other autoimmune diseases as well [17]. In 2024, a comprehensive review was published by Mosaddad et al. [18] on dental implants in patients with systemic diseases. Their review highlighted important aspects relevant to our case report: Systemic scleroderma might result in unfavorable dental occlusion and root resorption in cases of jaw deformity [19]. All organs are impacted by systemic scleroderma, including the oral system. Still, those with scleroderma who have lost teeth can consider dental implants [20]. In a case report [21], there were no radiological anomalies or pocket development surrounding the implants 24 months following the procedure. Oral rehabilitation might be feasible, but it's important to carefully consider the benefits, dangers, and planning of surgery [21]. After a 3-year follow-up, Zigdon et al. [22] discovered satisfactory oral hygiene and clinical characteristics without radiological signs of peri-implant bone loss. Implant-supported rehabilitation seems to be a feasible therapeutic option for individuals with scleroderma who have been on systemic steroids for a prolonged period [22]. Mosaddad et al.'s systematic review in 2023 [2] looked at data from 37 people with 153 implants who were diagnosed with scleroderma between the ages of 28 and 77. Based on case report studies, they reported a 100% survival rate; based on case series studies, it indicated an 89.2% survival rate. The majority of patients were female, and follow-up durations ranged from 1 to 10 years [2]. According to them, the rate of implant survival was not substantially influenced by scleroderma status. Implant-based therapy might not worsen overall morbidity or conflict with systemic medicines in scleroderma patients [2].

Because of the progressive nature of limited jaw opening in SS patients, screw-type implants were applied for the posterior areas of the current patient. Still, in such patients with limited mouth opening, cement-retained restorations provide more convenient access to the posterior regions of the oral cavity [12]. The main benefit of screw-retained restorations is allowing the operator to conveniently retrieve them, adjust the prosthodontic components, refasten the screws, and repair the fractured components without damaging the fixture or restoration and thus with less time and at lower cost compared to cement-retained restorations [13].

## 4. Conclusion

This report suggests that at least some SS patients might benefit from implant-based prosthodontic treatments, despite their systemic condition.

## Data Availability

This case report has no statistical data. The other data are already presented in the paper.
